# P-161. Real-world Outcomes of Patients Receiving Fecal Microbiota Transplant for Recurrent C. Difficile Infection

**DOI:** 10.1093/ofid/ofaf695.385

**Published:** 2026-01-11

**Authors:** Saher Siddiqui, Anita Shallal, Geehan Suleyman, Mayur Ramesh, Angela Ishak, Michael P Veve

**Affiliations:** Henry Ford Health, West Bloomfield, MI; Henry Ford Hospital, Detroit, Michigan; Henry Ford Health, West Bloomfield, MI; Henry Ford Hospital, Detroit, Michigan; Henry Ford Hospital, Detroit, Michigan; Eugene Applebaum College of Pharmacy and Health Sciences, Detroit, MI

## Abstract

**Background:**

Up to 30% of patients with *Clostridioides difficile* infection (CDI) recur after initial treatment. Fecal microbiota transplantation (FMT) restores gut microbiota and was highly effective in reducing recurrence of CDI (rCDI) in clinical trials, though real-world outcomes vary based on patient factors. This study evaluated outcomes of FMT, including rCDI and predictors of early FMT failure.

**Table 1.**
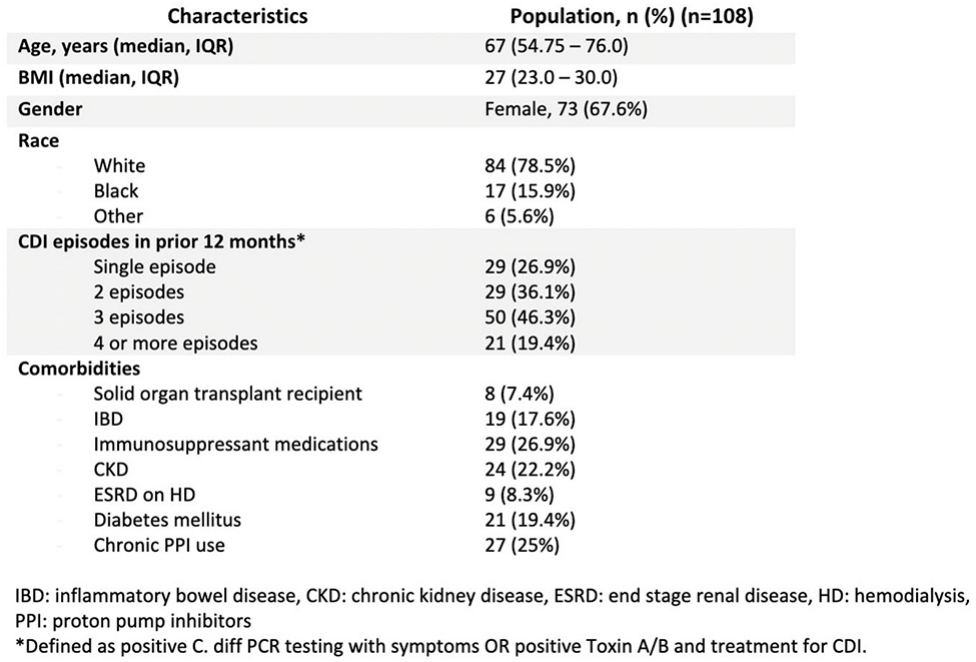
Patient demographics and primary infection characteristics Baseline demographics and number of reoccurrences prior to FMT.

**Table 2.**
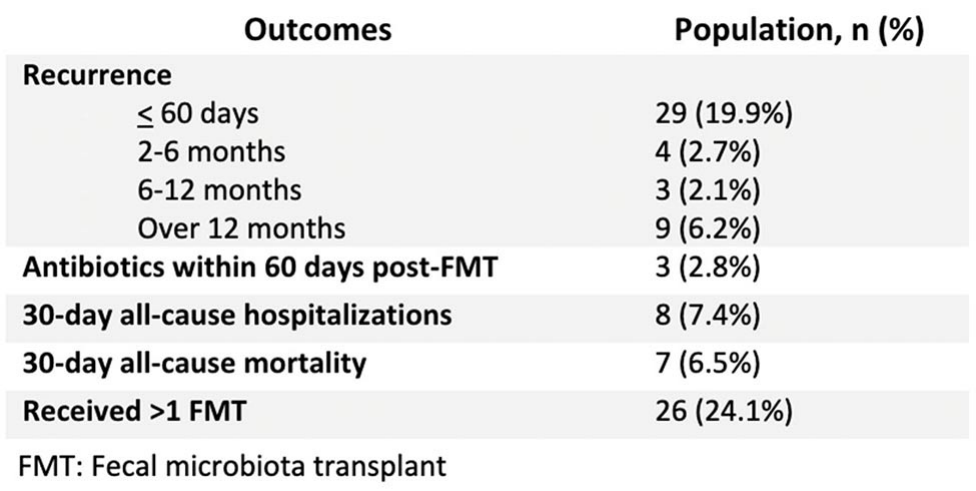
Recurrences, subsequent antibiotic use, and outcomes after FMT Clinical outcomes following FMT, including recurrence rates, post-procedure events, and need for repeat transplantation.

**Methods:**

Retrospective cohort study of adult outpatients who received FMT via donor stool enema prepared in accordance with institutional protocols for rCDI between 2018 and 2023 at Henry Ford Hospital. Patients with incomplete data were excluded. Collected data included demographics, comorbidities, prior CDI (pCDI) and rCDI, and subsequent antibiotic (abx) use. A multistep algorithm with enzyme immunoassay (EIA) for toxin A/B and glutamate dehydrogenase antigen followed by NAAT for discordant EIA results is utilized in our institution. Recurrence was defined as a positive stool test and treatment of CDI. The primary outcome was rCDI within 60 days post-FMT. Univariate logistic regression was conducted using R version 4.2.2 to screen potential predictors of recurrence within 60 days. Variables meeting a significance threshold of p ≤ 0.2 were subsequently entered into a multivariate logistic regression model to identify independent risk factors.

**Table 3.**
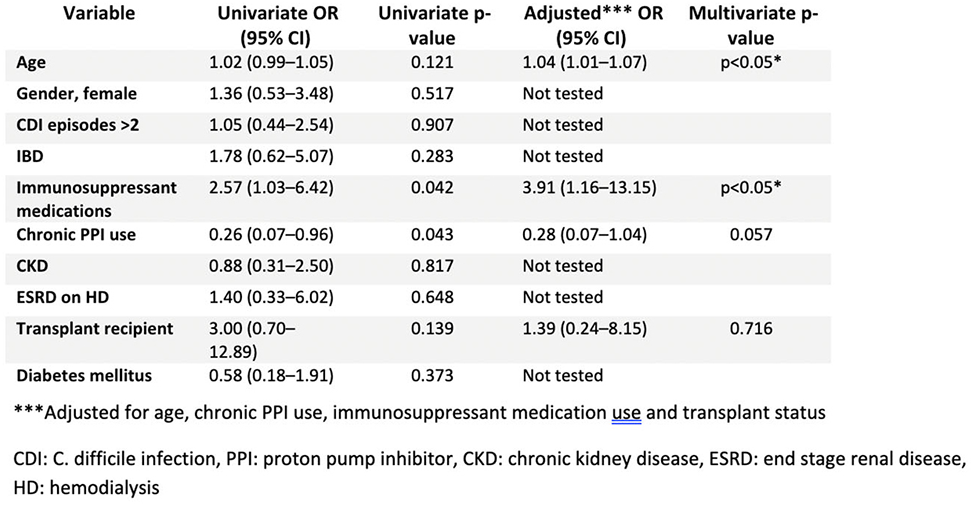
Univariate and multivariate analysis depicting the risk factors for CDI recurrence within 60 days after FMT Multivariable analysis of factors associated with 60-day CDI recurrence following FMT. Older age and use of immunosuppressant medications were independently associated with increased odds of recurrence.

**Results:**

Of 148 patients who received FMT, 108 were included with median age 67 years. Most were white (78.5%) females (67.6%) with >2 episodes of pCDI (65.7%) [Table 1]. Patients were rarely exposed to abx within 60 days of FMT. In most FMT patients, rCDI occurred within 12 months; of these, 29 (20%) within 60 days (Table 2). Age (p< 0.05) and receipt of immunosuppressant medications (p< 0.05) were associated with 60-day rCDI but not abx exposure or pCDI [Table 3]. Almost a quarter (24%) of patients received more than one FMT.

**Conclusion:**

In this large study of patients receiving FMT for rCDI, the 60-days recurrence rate was similar to that observed in earlier clinical trials, with older patients and those receiving immunosuppressant medications more likely to fail.

**Disclosures:**

Mayur Ramesh, MD, Citius Pharmaceuticals, Inc.: Grant/Research Support

